# How Frequent Are Eating Disturbances in the Population? Norms of the Eating Disorder Examination-Questionnaire

**DOI:** 10.1371/journal.pone.0029125

**Published:** 2012-01-18

**Authors:** Anja Hilbert, Martina de Zwaan, Elmar Braehler

**Affiliations:** 1 Integrated Research and Treatment Center AdiposityDiseases, Department of Medical Psychology and Medical Sociology, University of Leipzig Medical Center, Leipzig, Germany; 2 Clinics for Psychosomatics and Psychotherapy, Hannover Medical School, Hannover, Germany; 3 Department of Medical Psychology and Medical Sociology, University of Leipzig Medical Center, Leipzig, Germany; Paris Institute of Technology for Life, Food and Environmental Sciences, France

## Abstract

**Objective:**

The Eating Disorder Examination-Questionnaire (EDE-Q) is a self-report instrument assessing the specific psychopathology and key behaviors of eating disorders. This study sought to determine the prevalence of eating disturbances, and to provide psychometric properties and norms of the EDE-Q, in a representative German population sample.

**Methods:**

A total of 2520 individuals (1166 men, 1354 women) were assessed with the EDE-Q.

**Results:**

Eating disorder psychopathology was higher and most key behaviors were more prevalent in women than in men. Psychopathology declined with age ≥65 in both sexes, and showed a peak at age 55–64 in men. Overall, 5.9% of the women and 1.5% of the men revealed eating disturbances. The prevalence of eating disturbances decreased with age in women and was significantly higher in obese than in normal-weight individuals. Psychometric analyses showed favorable item characteristics. Internal consistencies of EDE-Q composite scores were ≥.80 for women and ≥.70 for men. The factor structure of the EDE-Q was partially reproduced. Sex- and age-specific population norms are reported.

**Discussion:**

This study provides population norms of the EDE-Q for both sexes and across the age range, demonstrates demographic variations in symptomatology, and reveals satisfactory psychometric properties. Further research is warranted on eating disturbances in older adults.

## Introduction

Eating disturbances are common health conditions in adults. They affect both women and men of different ages and weights [1], [2], and may present as full-blown eating disorders, predominantly in young females [3], [4]. Recently, an increase of eating disorder symptoms has been documented in both sexes and in those of higher age and body weight [1], but comprehensive information on eating disturbances across ages, sex, and weight status remains sparse [5].

Eating disturbances are associated with increased psychopathology, health problems, and impairment in quality of life [4], [6], [7]. These disturbances include a range of non-normative eating- or weight-related symptoms such as: binge eating, defined as eating an objectively large amount of food accompanied by a sense of loss of control over eating [8]; compensatory behaviors aimed at preventing weight gain (e.g., self-induced vomiting, laxative misuse); and attempts at restricting food intake, frequently motivated by concerns about eating, shape, or weight [7].

Reliable and valid instruments for assessment and diagnosis are key for the identification of eating disturbances. While diagnosing an eating disorder requires an interviewer's assessment [9], self-report questionnaires represent economic and non-intrusive approaches to assess eating disturbances. One well-established self-report questionnaire that allows for comprehensive assessment of eating disturbances is the Eating Disorder Examination-Questionnaire (EDE-Q) [9], [10], which was developed based on the Eating Disorder Examination (EDE), a semi-structured eating disorder interview [8], [11] considered the method of choice for eating disorder diagnosis and assessment [12]. Using similar operational definitions and time frames, the EDE-Q measures specific eating disorder psychopathology on four subscales, and facilitates assessment of diagnostically relevant behavioral features, such as binge eating, laxative misuse, excessive exercising, and fasting.

Numerous studies in adults with eating disturbances show that the EDE-Q composite indicators have good internal consistency [13], [14], temporal stability [13], [15], [16], high convergent validity [17]–[21], discriminant validity, and sensitivity to change [20], [22], [23]. The EDE-Q typically yields a high level of agreement with the EDE in the assessment of psychopathology, but more discrepancies occur in the assessment of key behavioral features, particularly binge eating [18], [19], [20], [21], [24]. Despite the overall favorable psychometric properties, evidence on the factorial validity is controversial [14], [25], and item statistics have not been reported systematically. Normative scores for young adult women [26], and female and male university students are available [27], [28], but population norms across ages and sex are lacking.

In this context, this study's primary goal was to determine the frequency of eating disorder psychopathology, behaviors, and eating disturbances in a representative sample of the German population. A secondary goal was to provide psychometric properties and population norms of the EDE-Q.

## Methods

### Recruitment and Sample

A representative sample of the German population was recruited in November and December 2009, with assistance by an independent agency specializing in market, opinion, and social research (USUMA, Berlin, Germany). A three-stage random sampling procedure was used to select (1) sample point regions from 258 regions that were determined based on representative data; (2) target households within sample point regions using a random route procedure; and (3) target persons within target households according to a kish selection grid. Inclusion criteria were age ≥14 years and fluent German.

Following this procedure, 4069 noninstitutionalized civilians were randomly selected from all German states. Of these, *N* = 2520 individuals participated in the assessment, corresponding to a response rate of 61.9% (398 [9.8%] households could not be reached; 539 [13.3%] refused to participate; 160 [3.9%] target persons could not be reached; 11 [0.3%] target persons were incapacitated; and 441 [10.8%] refused to participate). A maximum of three attempts was made to contact a target person. All participants were visited in-person, informed about the study procedures by a trained research assistant, and signed an informed consent prior to assessment (for minor participants, informed consent was additionally obtained from one parent). The ethical guidelines of the International Code of Marketing and Social Research Practise by the International Chamber of Commerce and the European Society for Opinion and Marketing Research were followed. The study was approved by the Ethics Committee of the University of Leipzig.

Sample characteristics are displayed in Table 1. The total study sample consisted of 1166 men (46.3%) and 1354 women (53.7%) with a mean age of 50.50 years (*SD* = 18.59; range 14–95 years) and a mean BMI of 25.25 kg/m^2^ (SD = 4.15; range 14.17–55.40 kg/m^2^), calculated from self-reported height and weight. Using the guidelines of the National Institutes of Health, 51.9% were underweight or normal weight (BMI<25.0 kg/m^2^; 2.1% underweight, i.e. BMI<18.0 kg/m^2^), 37.3% were overweight (BMI 25.0–29.9 kg/m^2^), and 10.8% were obese (BMI≥30.0 kg/m^2^). Further, 83.7% had <12 years of education, 56.1% had a household income of <EUR 2000, 52.1% were married, 19.9% lived in Eastern Germany, and 3.3% had a nationality other than German.

**Table 1:** Sociodemographic Characteristics (N = 2520).

		Women (N = 1354)	Men (N = 1166)
		N (%)	N (%)
Age, years	≤24	137 (10.1)	133 (11.4)
	25–34	161 (11.9)	132 (11.3)
	35–44	209 (15.4)	201 (17.2)
	45–54	238 (17.6)	198 (17.0)
	55–64	235 (17.4)	181 (15.5)
	65–74	211 (15.6)	232 (19.9)
	≥75	163 (12.0)	89 (7.6)
Weight Status	Underweight/Normal weight (<25.0 kg/m^2^)	769 (58.0)	518 (44.8)
	Overweight (25.0–29.9 kg/m^2^)	400 (30.2)	527 (45.6)
	Obesity (≥30.0 kg/m^2^)	157 (11.8)	111 (9.6)
Education	<12 years	1163 (85.9)	946 (81.1)
	≥12 years	191 (14.1)	220 (18.9)
Household	<EUR 2000	768 (58.1)	602 (53.8)
income	≥EUR 2000	554 (41.9)	518 (46.3)
Marital status	Married	664 (49.0)	650 (55.7)
	Single, divorced, widowed	690 (51.0)	516 (44.3)
Residence	Eastern part of Germany	265 (19.6)	237 (20.3)
	Western part of Germany	1089 (80.4)	929 (79.7)
Nationality	German	1319 (97.4)	1117 (95.8)
	Other	35 (2.6)	49 (4.2)

*Notes*. Calculation of % from valid cases (n).

### Eating Disorder Examination-Questionnaire

The authorized German translation of the EDE-Q 6.0 was performed by the first author and controlled by a retranslation procedure through a licensed translator. The EDE-Q includes 22 items allocated to the subscales of Restraint (RS) and Eating Concern (EC; 5 items each), both describing abnormalities in eating behavior, and Weight Concern (WC; 5 items) and Shape Concern (SC; 8 items), both measuring aspects of a negative body image. All items refer to the preceding 28 days, and frequency or intensity are rated on seven-point Likert scales (0 = *feature was absent* to 6 = *feature was present every day* or *to an extreme degree*). Mean subscale scores and a mean Global Score (GS) of overall eating disorder psychopathology were calculated. An empirically derived Global Score threshold ≥2.30 (versus <2.30) was used as an indicator of eating disturbances [19].

An additional six items measure diagnostically relevant information, e.g., number of episodes (or days) with objective bulimic episodes, and number of episodes of self-induced vomiting, laxative misuse, or driven exercising (i.e., compulsive exercising for controlling shape, weight, or amount of fat, or burning off calories). All key behavioral features were dichotomized for any occurrence (≥1 episode versus 0 episodes over the past 28 days) and regular occurrence (≥4 episodes versus <4 episodes over the past 28 days) [26], [27]. In addition, extreme dietary restriction was determined through the item Avoidance of Eating assessing days without eating anything during ≥8 waking hours in order to influence shape or weight. For extreme dietary restriction, a code ≥1 (i.e., ≥1 day over the past 28 days) was used as an indicator of any occurrence, and a code ≥3 (i.e., >12 days over the past 28 days) was used as an indicator of regular occurrence [26]. Any occurrence and regular occurrence of total key behaviors were determined.

An evaluation of the German translation of the EDE-Q 4.0 and 5.0 in N = 706 individuals with anorexia nervosa, bulimia nervosa, atypical eating disorders, restrained eating, and non-clinical as well as psychiatric comparison groups showed that the EDE-Q was internally consistent and stable, and had good convergent and discriminant validity, and sensitivity to change [29]. The factorial structure was partially confirmed.

### Data Analytic Plan

Distributions of EDE-Q subscale scores and Global Score as well as any or regular key behavioral features were analyzed using multi- and/or univariate Sex×Age General Linear Model analyses or Generalized Linear Model analyses (logit link function, binomial error distribution) for continuous or categorical variables, respectively. Univariate and post-hoc test results were only interpreted when significant higher-order effects were found. For the analysis of any or regular occurrence of key behavioral features, single key behaviors were analyzed only when effects were significant for total key behaviors. Partial η^2^ describing the proportion of total variability attributable to a factor was displayed for estimation of effect sizes when appropriate (η^2^: small: 0.01, medium: 0.06, large: 0.14) [30]. Sex- and age-specific percentiles were determined for the EDE-Q subscales and the Global Score. Distributions of eating disturbances by sociodemographic characteristics were determined in a multivariate logistic regression analysis on the Global Score ≥2.30 (versus <2.30). All sociodemographic characteristics (see Table 1) were simultaneously entered into the regression analysis. A Bonferroni corrected two-tailed α of .01 (.05 divided by 5) was applied to all tests because five sets of hypotheses were tested simultaneously (i.e., Sex×Age differences in EDE-Q composite scores, in the occurrence of key behavioral features, and in the occurrence of regular key behavioral features; and sex differences and other sociodemographic differences in eating disturbance).

For psychometric analyses, missing data, item descriptives, item difficulties [*p*_m_ = sum of item scores/(N * maximal item score)], corrected item-total correlations r_it_, and item homogeneity per subscale (i.e., average inter-item correlations) were determined, and item distributions were tested for normality using the Shapiro-Wilks normality test. Cronbach's α was used as a measure of internal consistency of composite EDE-Q indicators, and correlations between the subscales were computed as a measure of test homogeneity. The factor structure of the 22 items allocated to subscales was determined using Principal Components Analyses with orthogonal VARIMAX rotation (extraction criterion of λ>1 and scree test). All psychometric analyses were performed for women and men separately.

All analyses were performed using PASW 18.0.

## Results

### Primary Analysis: Distributions of Psychopathology, Key Behaviors, and Eating Disturbances

#### Eating disorder psychopathology

**Figure 1:**
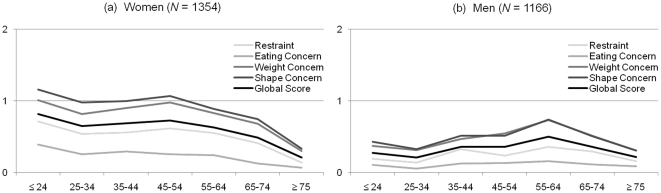
Subscale Scores and Global Scores of the Eating Disorder Examination-Questionnaire Across the Age Range (N = 2520). *Notes*. Scores ranging from 0 = *feature was absent* to 6 = *feature was present every day* or *to an extreme degree*. Higher values indicate greater psychopathology. Post-hoc analyses women: Restraint (RS), Weight Concern (WC), Shape Concern (SC), Global Score (GS) ages ≥75 lower than all other ages; Eating Concern (EC) age ≥75 lower than age ≤24 and 35–44, and age 65–74 lower than age ≤24; WC and GS age 65–74 lower than age ≤24 (all p<.01). Post-hoc analyses men: WC, SC, and GS age 55–64 greater than age ≤24, 25–34, and ≥75 (all p<.01).

Figure 1 depicts the EDE-Q composite indicators across the age range in both sexes. A multivariate analysis of subscale scores and the Global Score by sex and age showed significant main and interaction effects [sex: F(4, 2495) = 25.54; age: F(24, 8705) = 3.43; Sex×Age: F(24, 8705) = 2.05, all p<.01], as did univariate analyses (all p<.05). Women displayed higher values on all EDE-Q subscales and the Global Score than men, with small effect sizes (0.02≤η^2^≤0.04). While women showed decreased subscale scores and Global Score at age ≥65 years, men had increased Weight Concern, Shape Concern, and Global Score at age 55–64 (all p<.01; see Figure 1 for post-hoc tests). Significant sex-specific variations by age had small to medium effect sizes (0.02≤η^2^≤0.10). Based on these analyses, sex- and age-specific percentile ranks were displayed for age groups of men and women homogenous with regard to eating disorder psychopathology (Table S1).

#### Key behavioral features

As displayed in Table 2, more women than men reported any occurrence of key behaviors over the past 28 days, specifically laxative misuse and extreme dietary restriction (all p<.01). Few significant variations were found by age in any occurrence of key behaviors, and Sex×Age differences failed to reach the corrected significance level (age: χ^2^(6, N = 2519) = 17.58, p<.01; Sex×Age: χ^2^(6, N = 2519) = 14.75, p = .02). Key behavioral features were less prevalent at age ≥75 than at age ≤55 (post-hoc p<.01). More specifically, fasting declined at age ≥65 when compared to younger age groups (age: χ^2^(6, N = 2512) = 25.32, p<.01, post-hoc p<.01; Sex×Age: χ^2^(6, N = 2512) = 14.79, p = .02). Regular occurrence of key behavioral features did not show any variations in Sex×Age analyses (all p>.01).

**Table 2:** Key Behavioral Features of the Eating Disorder Examination-Questionnaire (N = 2520).

	Women		Men			
	(N = 1354)		(N = 1166)			
*Any occurrence past 28 days*	n	%	n	%	Wald χ^2^ (df = 1)	p
Key behaviors (total)	235	17.4	153	13.1	8.62	<.01*
Objective bulimic episodes	57	4.2	49	4.2	0.00	1.00
Self-induced vomiting	18	1.3	5	0.4	5.06	.02
Laxative misuse	33	2.4	9	0.8	9.53	<.01*
Driven exercising^a^	50	3.7	41	3.5	0.05	.82
Extreme dietary restriction^b^	161	11.9	89	7.7	12.59	<.01*
*Regular occurrence past 28 days (≥4 episodes)*						
Key behaviors (total)	74	5.5	45	3.9	3.57	.06
Objective bulimic episodes	19	1.4	11	0.9	1.10	.29
Self-induced vomiting	4	0.3	1	0.1	1.23	.27
Laxative misuse	10	0.7	2	0.2	3.57	.06
Driven exercising^a^	26	1.9	23	2.0	0.10	.92
Extreme dietary restriction^b^	33	2.4	16	1.4	3.64	.06

a Driven exercising was defined as compulsive exercising for controlling shape, weight, or amount of fat, or burning off calories.

b Extreme dietary restriction was determined through the item Avoidance of Eating assessing days without eating anything in order to influence shape or weight, coded ≥1 (i.e., ≥1 day over the past 28 days) for determining any occurrence, or coded ≥3 (i.e., >12 days over the past 28 days) for determining regular occurrence.

*p<.01.

#### Eating disturbances

Using a Global Score of 2.30, 3.9% of individuals revealed eating disturbances – women were five times more likely to reveal eating disturbances than men [5.9% versus 1.5%, Exp(B) = 4.85, 95% CI 2.76–8.50, χ^2^(1, N = 2519) = 30.28, p<.01]. As displayed in Table S2, women age ≤24 years were more likely to show eating disturbances than women at all other age groups (all p<.01). Compared to underweight or normal weight women, overweight women had a 2-fold higher probability, and obese women an 11-fold higher probability, of eating disturbances (all p<.01). In men, obese men showed a 20-fold higher probability of eating disturbances than underweight or normal weight men [p<.01; total sample: 12-fold increased probability in obese individuals; Exp(B) = 11.53, 95% CI 6.49–20.49, χ^2^(1, N = 2519) = 69.38, p<.01].

### Secondary Analysis: Psychometric Properties

#### Item analysis

For all items, missing data were low with ≤0.5% of missing item responses per subscale (women: 0.5±0.0%; men: 0.4±0.1%), and composite indicators could be determined for all participants. Overall, 77.1% of item codes per subscale were 0, indicating absence of eating disorder psychopathology (women: 72.9±13.9%; men: 82.0±20.5%), while 4.6% of item codes were ≥4, which is a theoretical threshold of eating disorders (women: 6.3±4.5%; men: 2.9±2.2%) [8]. All items were positively skewed and had a high kurtosis, and item distributions deviated significantly from normality (all p<.01; Table S3). Item difficulties were high, with a low probability of scores >0 (women: .02≤p_m_≤.23; men: .01≤p_m_≤.15). Corrected item-total correlations were in the middle to upper range (women: .48≤r_it_≤.82; men: .37≤r_it_≤.77). Homogeneity of items allocated to a subscale was mostly optimal for men and slightly higher in women (mean inter-item correlation for women/men: RS, r = .53/.46; EC, .48/.32; WC, .47/.35; SC, .53/.44: GS, .45/33).

Missing data for key behavioral features were low with ≤0.3% of missing responses per item (women: 0.1±0.1%; men: 0.3±0.1%). Key behavioral features were strongly positively skewed and had a high kurtosis (women/men: skewness, 3.50–21.27/5.85–33.84; kurtosis, 15.43–573.58/41.27–1147.10).

#### Reliability and homogeneity

Internal consistency of subscales and Global Score was acceptable to excellent (Cronbach's α for women/men: RS, .84/.80; EC, .81/.70; WC, .80/.72; SC, .90/.86: GS, .94/.91). Correlations between subscales were mostly high (women: .61≤r≤.93; men: .46≤r≤.89).

#### Factorial validity

For the female and male subsamples, Principal Component Analyses resulted in largely similar three-factor solutions; thus, results for the total sample analyses will be reported. For the total sample, the three-factor solution explained 62.5% of the total item variance (Table S4). Factor I was composed by 5 Shape Concern items, 3 Weight Concern items, and 1 Eating Concern item focused on attitudinal and cognitive aspects of a negative body image. Factor II included 3 Restraint items, 2 Shape Concern items, and 1 Weight Concern item concerned with behavioral and motivational aspects of dietary restriction. Factor III was composed by 4 Eating Concern items, 2 Restraint items, and 1 Shape/Weight Concern item focused on cognitive symptoms of eating disturbances. Eleven items had substantial cross-loadings (≥.30).

## Discussion

The EDE-Q is a prominent questionnaire that is increasingly being utilized internationally for the comprehensive assessment of the specific psychopathology and key behavioral features of eating disorders. Using the EDE-Q in a representative German sample, distributional analyses revealed an overall higher level of eating disorder psychopathology in women than in men; however, with small effect sizes. Likewise, current research has found similar eating disorder symptoms at a slightly to moderately higher degree in women [28], [31], [32]. In both sexes, subscale scores and Global Scores decreased at age ≥65 years. This result extends the existing evidence that eating disorder psychopathology such as a negative body image that are present in older women [33], [34], are also present in older men. In addition, a late life decrease of eating disorder psychopathology was found. This decrease parallels a decline in prevalence rates of mental disorders [35], [36] that may be attributable to late life-specific perceptions, conditions, or attitudes. For men, a peak of Weight Concern, Shape Concern, and the Global Score at ages 55–64 was notable, and presumably related to increasing levels of obesity in this age group [37], [38] and a concomitant desire to lose weight. Based on these analyses, population norms were reported for homogeneous age groups of younger, middle-aged, and older men and women. To our knowledge, these are the EDE-Q's first population norms for both sexes and across the adult age range.

For the key behavioral features, women endorsed total key behaviors, laxative misuse, and extreme dietary restriction more frequently, but reported binge eating, vomiting, and driven exercising at similar rates to men. Of note, sex differences on the regular occurrence of any key behavioral feature were only trendwise significant, including regular laxative misuse and extreme dietary restriction (all p = .06). In recent epidemiological studies, few differences between the sexes on key behavioral features have been found, with the exception of compensatory behaviors [28], [31]. Some evidence, albeit inconsistent, suggests that disordered eating behaviors in men have increased over the past decades at least as much as in women [31]. The question of whether prevalence rates of specific eating disorders converge [3] needs to be further examined by using validated instruments. Extreme dietary restriction decreased over age in both sexes, which is consistent with previous research [1], [2]. However, the absence of age effects on the occurrence of binge eating or purging, and on the regular occurrence of all key behavioral features, is in contrast to previous population surveys that, however, used other definitions and/or interview-based assessment [1], [2], [39].

Using a validated threshold of the EDE-Q Global Score with high sensitivity and specificity [19], eating disturbances were identified in 5.9% of women and 1.5% of men. This cut-off score, focusing on cognitive and behavioral aspects of eating disorders, was used to identify clinically relevant eating disturbances. The EDE-Q is not designed to diagnose specific eating disorders, and the self-report assessment of diagnostically relevant behaviors frequently diverges from expert ratings [18], [19], [20], [21], [24]. Because self-report may involve an overestimation of psychopathology, the rates of eating disturbances in this study are equal to or higher than current interview-based lifetime prevalence rates of eating disorders [3], [40]. In this study, women had a higher probability of eating disturbances than men. Consistent with epidemiological data [41], young women ≤24 years were significantly more prone to eating disturbances than older men and women. Of note, obese participants of both sexes had an 11 to 20 times higher risk of eating disturbances compared to normal weight participants. The association between obesity and eating disorder psychopathology has been documented previously [42], [43]. Nevertheless, the magnitude of effect underscores the substantial mental suffering associated with obesity. Consistent with some previous research, no association was observed between eating disturbances and household income or educational level [44].

### Psychometric Properties

Concerning the psychometric properties of the EDE-Q, item analyses showed that the EDE-Q was well-received by the participants, which has been found previously in young adults [26]–[28]. The focus of the EDE-Q on revealing higher-level eating disorder psychopathology was reflected in high item difficulties, and positively skewed and sharply peaked item distributions. Based on favorable corrected item-total correlations and item homogeneity, internal consistencies of the EDE-Q subscales and the Global Score were good for women and at least acceptable for men, which is in accordance with literature on younger persons [13], [14], [28].

Suitability of the EDE-Q for assessment of eating disorder psychopathology in both sexes was also corroborated by the EDE-Q's factorial validity. In regards to previous evidence on the suitability of the EDE-Q in women with and without eating disturbances [14], [29], for both the female and male subsamples, Restraint and Eating Concern were mostly reproduced, and Weight Concern and Shape Concern items largely combined into one factor. Of note, however, are numerous high cross-loadings and intercorrelations among the subscales. When interpreting subscale profiles, it is therefore necessary to consider that subscales are not independent from another. This overlap between subscales is inherent to the construction of the EDE that focused more on validity than on homogeneity or independence of subscales [8], [11], and thus facilitates to capture the interrelated nature of eating disorder psychopathology. Nevertheless, as Weight and Shape Concern are highly related constructs, a combined Weight/Shape Concern Scale would correspond more to factor analytical results than separate subscales. Combining these scales was also supported by confirmatory factor analysis [25].

### Strengths and Limitations

Strengths of the present study include the use of a large, population-based sample drawn in order to be representative for sex and age. Compared to the data of the Federal Statistical Office from 2008 and 2009, our sample included slightly fewer males (46.3% versus 49.0%) and more participants of older ages (45–74 years; 61.4% versus 47.7%). In order to avoid potential sampling effects, all analyses were sex-specific or sex- and age-specific. In addition, all analyses were repeated after weighting data for age, sex, and state of residency, yielding similar results as the analyses of raw data. Finally, self-report leads to underestimation of overweight and obesity prevalence rates [41], which may have lessened the effects of greater eating disorder psychopathology in obese individuals, compared to normal weight individuals.

### Conclusion

The current study demonstrates sex- and age-specific variations in symptomatology. It also provides psychometric properties, including population norms of the EDE-Q, for both sexes and across the adult age range. As one of the first studies to adopt a lifespan perspective on eating disorder psychopathology in a cross-sectional design, the results nevertheless underline the need for longitudinal research on the impact of advancing age in the eating disorder psychopathology of both women and men.

## Supporting Information

Table S1 Sex- and Age-Specific Norms of the Eating Disorder Examination-Questionnaire (N = 2520).
(DOC)

Table S2 Distribution of Eating Disturbances as Determined by the Eating Disorder Examination-Questionnaire Global Score ≥2.30 (N = 2520).
(DOC)

Table S3 Item Characteristics of the Eating Disorder Examination-Questionnaire (N = 2520).
(DOC)

Table S4 Principal Components Analysis of the Eating Disorder Examination-Questionnaire Items (N = 2520).
(DOC)
