# Supplementary material for: How Frequent Are Eating Disturbances in the Population? Norms of the Eating Disorder Examination-Questionnaire
Source: PLoS One. 2012 Jan 18;7(1):e29125. doi: 10.1371/journal.pone.0029125 (PMC3261137; doi:10.1371/journal.pone.0029125)
Supplement: Table S1 — Sex- and Age-Specific Norms of the Eating Disorder Examination-Questionnaire (N = 2520). (DOC) [file pone.0029125.s001.doc]

Table S1. Sex- and Age-Specific Norms of the Eating Disorder Examination-Questionnaire (N = 2520).

| Women (N = 1354) | | | | | | | | | | | | | | | | | |  |
| --- | --- | --- | --- | --- | --- | --- | --- | --- | --- | --- | --- | --- | --- | --- | --- | --- | --- | --- |
| ≤ 44 Years | | | | | | 45-64 Years | | | | | | ≥ 65 Years | | | | | | |
| Perc. | RS | EC | WC | SC | GS | Perc. | RS | EC | WC | SC | GS | Perc. | RS | EC | WC | SC | GS | |
| 1-25 | - | - | - | - | - | 1-25 | - | - | - | - | - | 1-25 | - | - | - | - | - | |
| 30 | - | - | - | 0.13 | 0.06 | 30 | - | - | - | - | 0.05 | 30 | - | - | - | - | - | |
| 35 | - | - | - | 0.25 | 0.14 | 35 | - | - | - | 0.13 | 0.10 | 35 | - | - | - | - | - | |
| 40 | - | - | 0.20 | 0.38 | 0.19 | 40 | - | - | 0.20 | 0.25 | 0.16 | 40 | - | - | - | - | - | |
| 45 | - | - | 0.23 | 0.38 | 0.29 | 45 | - | - | 0.40 | 0.38 | 0.27 | 45 | - | - | - | - | 0.03 | |
| 50 | - | - | 0.40 | 0.63 | 0.34 | 50 | - | - | 0.40 | 0.50 | 0.36 | 50 | - | - | - | 0.13 | 0.11 | |
| 55 | - | - | 0.60 | 0.75 | 0.45 | 55 | - | - | 0.60 | 0.63 | 0.44 | 55 | - | - | 0.20 | 0.25 | 0.16 | |
| 60 | 0.20 | - | 0.60 | 0.88 | 0.52 | 60 | 0.20 | - | 0.80 | 0.88 | 0.53 | 60 | - | - | 0.40 | 0.38 | 0.23 | |
| 65 | 0.40 | 0.20 | 1.00 | 1.00 | 0.66 | 65 | 0.40 | 0.20 | 1.00 | 1.00 | 0.63 | 65 | - | - | 0.40 | 0.50 | 0.30 | |
| 70 | 0.60 | 0.20 | 1.20 | 1.25 | 0.79 | 70 | 0.40 | 0.20 | 1.20 | 1.25 | 0.81 | 70 | - | - | 0.60 | 0.70 | 0.41 | |
| 75 | 0.80 | 0.20 | 1.40 | 1.50 | 1.00 | 75 | 0.60 | 0.20 | 1.40 | 1.50 | 1.03 | 75 | - | - | 0.80 | 0.88 | 0.49 | |
| 80 | 1.20 | 0.40 | 1.60 | 1.88 | 1.24 | 80 | 1.00 | 0.40 | 1.80 | 1.88 | 1.28 | 80 | 0.36 | 0.20 | 1.00 | 1.00 | 0.58 | |
| 85 | 1.40 | 0.60 | 2.00 | 2.38 | 1.60 | 85 | 1.60 | 0.60 | 2.20 | 2.25 | 1.53 | 85 | 0.60 | 0.20 | 1.20 | 1.25 | 0.82 | |
| 90 | 2.00 | 1.00 | 2.80 | 3.00 | 1.95 | 90 | 2.20 | 0.80 | 2.60 | 2.75 | 1.90 | 90 | 1.18 | 0.40 | 1.72 | 1.88 | 1.21 | |

Table S1 (cont.)

| Women (N = 1354) | | | | | | | | | | | | | | | | | |  |
| --- | --- | --- | --- | --- | --- | --- | --- | --- | --- | --- | --- | --- | --- | --- | --- | --- | --- | --- |
| ≤ 44 Years | | | | | | 45-64 Years | | | | | | ≥ 65 Years | | | | | | |
| Perc. | RS | EC | WC | SC | GS | Perc. | RS | EC | WC | SC | GS | Perc. | RS | EC | WC | SC | GS | |
| 95 | 2.80 | 1.80 | 3.60 | 4.00 | 2.79 | 95 | 3.07 | 1.40 | 3.20 | 3.66 | 2.54 | 95 | 1.69 | 0.80 | 2.40 | 2.75 | 1.72 | |
| 99 | 4.75 | 3.38 | 4.80 | 5.25 | 4.19 | 99 | 4.65 | 2.80 | 4.60 | 5.00 | 3.88 | 99 | 4.80 | 1.20 | 3.65 | 3.91 | 2.67 | |
| Men (N = 1166) | | | | | | | | | | | | | | | | | |  |
| ≤ 44 Years | | | | | | 45-64 Years | | | | | | ≥ 65 Years | | | | | | |
| Perc. | RS | EC | WC | SC | GS | Perc. | RS | EC | WC | SC | GS | Perc. | RS | EC | WC | SC | GS | |
| 1-35 | - | - | - | - | - | 1-35 | - | - | - | - | - | 1-35 | - | - | - | - | - | |
| 40 | - | - | - | - | - | 40 | - | - | - | - | 0.06 | 40 | - | - | - | - | - | |
| 45 | - | - | - | - | 0.05 | 45 | - | - | 0.20 | 0.13 | 0.13 | 45 | - | - | - | - | - | |
| 50 | - | - | - | 0.13 | 0.08 | 50 | - | - | 0.20 | 0.25 | 0.18 | 50 | - | - | - | - | 0.05 | |
| 55 | - | - | - | 0.25 | 0.14 | 55 | - | - | 0.40 | 0.38 | 0.24 | 55 | - | - | 0.20 | 0.13 | 0.10 | |
| 60 | - | - | 0.20 | 0.25 | 0.19 | 60 | - | - | 0.40 | 0.50 | 0.31 | 60 | - | - | 0.24 | 0.25 | 0.16 | |
| 65 | - | - | 0.40 | 0.38 | 0.23 | 65 | - | - | 0.60 | 0.63 | 0.39 | 65 | - | - | 0.40 | 0.25 | 0.21 | |
| 70 | - | - | 0.40 | 0.50 | 0.29 | 70 | 0.20 | - | 0.80 | 0.75 | 0.51 | 70 | - | - | 0.60 | 0.38 | 0.30 | |

Table S1 (cont.)

| Men (N = 1166) | | | | | | | | | | | | | | | | | |  |
| --- | --- | --- | --- | --- | --- | --- | --- | --- | --- | --- | --- | --- | --- | --- | --- | --- | --- | --- |
| ≤ 44 Years | | | | | | 45-64 Years | | | | | | ≥ 65 Years | | | | | | |
| Perc. | RS | EC | WC | SC | GS | Perc. | RS | EC | WC | SC | GS | Perc. | RS | EC | WC | SC | GS | |
| 75 | - | - | 0.60 | 0.63 | 0.38 | 75 | 0.20 | 0.20 | 1.00 | 1.00 | 0.63 | 75 | - | - | 0.60 | 0.50 | 0.41 | |
| 80 | 0.20 | 0.20 | 0.80 | 0.75 | 0.50 | 80 | 0.40 | 0.20 | 1.20 | 1.13 | 0.76 | 80 | 0.20 | - | 0.80 | 0.75 | 0.54 | |
| 85 | 0.40 | 0.20 | 1.00 | 1.00 | 0.65 | 85 | 0.60 | 0.20 | 1.40 | 1.50 | 0.98 | 85 | 0.40 | 0.20 | 1.20 | 1.13 | 0.74 | |
| 90 | 0.88 | 0.40 | 1.40 | 1.38 | 0.85 | 90 | 1.20 | 0.40 | 2.00 | 1.75 | 1.16 | 90 | 0.80 | 0.40 | 1.40 | 1.38 | 1.06 | |
| 95 | 1.54 | 0.60 | 1.80 | 2.00 | 1.25 | 95 | 2.00 | 1.00 | 2.40 | 2.38 | 1.76 | 95 | 1.20 | 0.60 | 2.18 | 2.11 | 1.38 | |
| 99 | 3.94 | 1.27 | 2.80 | 3.25 | 2.53 | 99 | 3.00 | 1.80 | 4.16 | 4.07 | 2.56 | 99 | 4.44 | 1.71 | 3.36 | 4.23 | 3.00 | |

*Notes*. RS, Restraint Scale; EC, Eating Concern; WC, Weight Concern; SC; Shape Concern; GS; Global Score.
