# Supplementary material for: How Frequent Are Eating Disturbances in the Population? Norms of the Eating Disorder Examination-Questionnaire
Source: PLoS One. 2012 Jan 18;7(1):e29125. doi: 10.1371/journal.pone.0029125 (PMC3261137; doi:10.1371/journal.pone.0029125)
Supplement: Table S2 — Distribution of Eating Disturbances as Determined by the Eating Disorder Examination-Questionnaire Global Score ≥2.30 (N = 2520). (DOC) [file pone.0029125.s002.doc]

Table S2. Distribution of Eating Disturbances as Determined by the Eating Disorder Examination-Questionnaire Global Score ≥ 2.30 (N = 2520).

|  | Women (N = 1354) | | | | Men (N = 1166) | | | |
| --- | --- | --- | --- | --- | --- | --- | --- | --- |
|  | % | Exp(B) | 95% CI | χ^2^(df = 1) | % | Exp(B) | 95% CI | χ^2^(df = 1) |
| Age |  |  |  |  |  |  |  |  |
| ≤ 24 | 13.9 | 1.00 |  |  | 1.5 | 1.00 |  |  |
| 25-34 | 5.6 | 0.29 | 0.12-0.72 | 7.16* | 0.0 | 0.00 | 0.00- | 0.00 |
| 35-44 | 7.2 | 0.29 | 0.12-0.70 | 7.69* | 1.5 | 1.56 | 0.14-17.97 | 0.13 |
| 45-54 | 6.3 | 0.22 | 0.09-0.52 | 11.77* | 2.0 | 1.65 | 0.15-18.42 | 0.16 |
| 55-64 | 6.8 | 0.19 | 0.08-0.47 | 13.04* | 1.7 | 1.11 | 0.09-13.74 | 0.01 |
| 65-74 | 1.9 | 0.04 | 0.01-0.15 | 22.30* | 1.7 | 1.66 | 0.14-20.29 | 0.16 |
| ≥ 75 | 1.2 | 0.05 | 0.01-0.24 | 14.30* | 1.1 | 0.94 | 0.05-19.56 | 0.00 |
| Weight Status |  |  |  |  |  |  |  |  |
| Underweight/Normal weight (< 25.0 kg/m^2^) | 3.6 | 1.00 |  |  | 0.4 | 1.00 |  |  |
| Overweight (25.0-29.9 kg/m^2^) | 5.0 | 2.44 | 1.28-4.63 | 7.40* | 1.1 | 2.30 | 0.42-12.75 | 0.91 |
| Obesity (> 30.0 kg/m^2^) | 19.1 | 10.93 | 5.73-20.87 | 52.58* | 8.1 | 20.02 | 3.91-102.60 | 12.91* |
| Education |  |  |  |  |  |  |  |  |
| < 12 years | 5.9 | 1.00 |  |  | 1.7 | 1.00 |  |  |
| > 12 years | 6.3 | 1.14 | 0.57-2.29 | 0.14 | 0.5 | 0.38 | 0.05-3.07 | 0.83 |

Table 3 (cont.)

|  | Women (N = 1354) | | | | Men (N = 1166) | | | |
| --- | --- | --- | --- | --- | --- | --- | --- | --- |
|  | % | Exp(B) | 95% CI | χ^2^(df = 1) | % | Exp(B) | 95% CI | χ^2^(df = 1) |
| Household income |  |  |  |  |  |  |  |  |
| < EUR 2000 | 5.0 | 1.00 |  |  | 2.0 | 1.00 |  |  |
| ≥ EUR 2000 | 7.4 | 1.21 | 0.69-2.10 | 0.44 | 0.8 | 0.74 | 0.19-2.88 | 0.19 |
| Marital status |  |  |  |  |  |  |  |  |
| Married | 6.0 | 1.00 |  |  | 1.1 | 1.00 |  |  |
| Single, divorced, widowed | 5.8 | 0.90 | 0.50-1.64 | 0.12 | 1.9 | 2.24 | 0.66-7.62 | 1.66 |
| Residence |  |  |  |  |  |  |  |  |
| Western part of Germany | 6.1 | 1.00 |  |  | 1.1 | 1.00 |  |  |
| Eastern part of Germany | 5.3 | 1.07 | 0.55-2.07 | 0.04 | 3.0 | 2.22 | 0.75-6.58 | 2.07 |
| Nationality |  |  |  |  |  |  |  |  |
| German | 5.8 | 1.00 |  |  | 1.5 | 1.00 |  |  |
| Other | 8.6 | 1.12 | 0.32-3.98 | 0.03 | 0.0 | 0.00 | 0.00- | 0.00 |

*Notes.* Multivariate logistic regression analyses, Wald χ^2^, 95% CI (confidence interval).

*p < .01
