# Supplementary material for: How Frequent Are Eating Disturbances in the Population? Norms of the Eating Disorder Examination-Questionnaire
Source: PLoS One. 2012 Jan 18;7(1):e29125. doi: 10.1371/journal.pone.0029125 (PMC3261137; doi:10.1371/journal.pone.0029125)
Supplement: Table S3 — Item Characteristics of the Eating Disorder Examination-Questionnaire (N = 2520). (DOC) [file pone.0029125.s003.doc]

Table S3. Item Characteristics of the Eating Disorder Examination-Questionnaire (N = 2520).

|  | Women (N = 1354) | | | | | | Men (N = 1166) | | | | | |
| --- | --- | --- | --- | --- | --- | --- | --- | --- | --- | --- | --- | --- |
|  | M | SD | Skewness | Kurtosis | p_m_ | r_it_ | M | SD | Skewness | Kurtosis | p_m_ | r_it_ |
| *Restraint Scale* | 0.51 | 1.20 | 2.52 | 6.65 | 0.08 | .68 | 0.25 | 0.86 | 3.92 | 19.07 | 0.04 | .61 |
| Restraint over eating | 0.75 | 1.56 | 2.36 | 4.71 | 0.12 | .77 | 0.39 | 1.16 | 3.63 | 13.35 | 0.07 | .65 |
| Avoidance of eating | 0.21 | 0.73 | 4.63 | 24.78 | 0.04 | .54 | 0.13 | 0.58 | 6.16 | 46.84 | 0.02 | .52 |
| Food avoidance | 0.64 | 1.35 | 2.56 | 6.29 | 0.11 | .76 | 0.29 | 0.90 | 4.35 | 21.50 | 0.05 | .70 |
| Dietary rules | 0.70 | 1.51 | 2.41 | 5.05 | 0.12 | .74 | 0.33 | 1.03 | 3.96 | 16.52 | 0.06 | .68 |
| Empty stomach | 0.23 | 0.85 | 4.79 | 25.18 | 0.04 | .52 | 0.13 | 0.61 | 6.74 | 52.57 | 0.02 | .49 |
| *Eating Concern Scale* | 0.23 | 0.71 | 4.06 | 20.93 | 0.04 | .62 | 0.12 | 0.47 | 4.72 | 29.96 | 0.02 | .46 |
| Preoccupation with food, eating or calories | 0.23 | 0.76 | 4.48 | 23.86 | 0.04 | .60 | 0.13 | 0.56 | 6.83 | 58.82 | 0.02 | .49 |
| Fear of losing control over eating | 0.25 | 0.89 | 4.45 | 21.41 | 0.04 | .63 | 0.11 | 0.52 | 6.98 | 60.21 | 0.02 | .49 |
| Eating in secret | 0.09 | 0.37 | 5.20 | 38.24 | 0.02 | .50 | 0.07 | 0.30 | 5.03 | 29.95 | 0.02 | .37 |
| Guilt about eating | 0.38 | 0.85 | 3.18 | 12.77 | 0.06 | .62 | 0.18 | 0.54 | 4.43 | 27.64 | 0.03 | .46 |
| Social eating | 0.18 | 0.70 | 5.02 | 29.08 | 0.03 | .74 | 0.09 | 0.42 | 6.58 | 57.67 | 0.01 | .48 |
| *Weight Concern Scale* | 0.80 | 1.39 | 1.66 | 2.48 | 0.13 | .62 | 0.49 | 1.07 | 2.09 | 5.10 | 0.08 | .50 |
| Preoccupation with shape or weight^a^ | 0.23 | 0.75 | 4.15 | 20.30 | 0.04 | .48 | 0.11 | 0.55 | 6.71 | 53.95 | 0.02 | .39 |
| Desire to lose weight | 1.01 | 1.81 | 1.87 | 2.22 | 0.17 | .66 | 0.51 | 1.26 | 3.14 | 9.88 | 0.08 | .55 |

Table S2 (cont.)

|  | Women (N = 1354) | | | | | | Men (N = 1166) | | | | | |
| --- | --- | --- | --- | --- | --- | --- | --- | --- | --- | --- | --- | --- |
|  | M | SD | Skewness | Kurtosis | p_m_ | r_it_ | M | SD | Skewness | Kurtosis | p_m_ | r_it_ |
| Importance of weight | 0.74 | 1.29 | 2.00 | 3.77 | 0.12 | .67 | 0.47 | 1.01 | 2.53 | 6.86 | 0.08 | .57 |
| Reaction to prescribed weighing | 0.68 | 1.35 | 2.23 | 4.42 | 0.11 | .54 | 0.45 | 1.07 | 2.86 | 8.70 | 0.08 | .39 |
| Dissatisfaction with weight | 1.35 | 1.76 | 1.25 | .56 | 0.22 | .70 | 0.90 | 1.46 | 1.81 | 2.65 | 0.15 | .58 |
| *Shape Concern Scale* | 0.89 | 1.49 | 1.70 | 2.58 | 0.15 | .70 | 0.50 | 1.09 | 2.37 | 6.75 | 0.08 | .63 |
| Flat stomach | 0.92 | 1.82 | 2.02 | 2.72 | 0.15 | .53 | 0.47 | 1.26 | 3.27 | 10.45 | 0.08 | .46 |
| Preoccupation with shape or weight^a^ | 0.23 | 0.74 | 4.15 | 20.30 | 0.04 | .47 | 0.11 | 0.55 | 6.71 | 53.95 | 0.02 | .45 |
| Fear of weight gain | 0.76 | 1.57 | 2.32 | 4.45 | 0.13 | .70 | 0.37 | 1.09 | 3.84 | 15.29 | 0.06 | .57 |
| Feelings of fatness | 0.94 | 1.69 | 1.99 | 2.95 | 0.16 | .78 | 0.48 | 1.17 | 3.20 | 10.79 | 0.08 | .72 |
| Importance of shape | 0.79 | 1.33 | 1.88 | 3.07 | 0.13 | .67 | 0.52 | 1.06 | 2.30 | 5.28 | 0.09 | .52 |
| Dissatisfaction with shape | 1.39 | 1.71 | 1.20 | .53 | 0.23 | .77 | 0.84 | 1.34 | 1.84 | 3.00 | 0.14 | .67 |
| Discomfort seeing body | 1.12 | 1.56 | 1.50 | 1.59 | 0.19 | .82 | 0.65 | 1.16 | 2.15 | 4.68 | 0.11 | .77 |
| Avoidance of exposure | 0.97 | 1.52 | 1.73 | 2.31 | 0.16 | .76 | 0.54 | 1.08 | 2.46 | 6.48 | 0.09 | .73 |
| *Global Score* | 0.67 | 1.26 | 2.06 | 4.76 | 0.10 | .66 | 0.36 | 0.91 | 2.50 | 7.69 | 0.06 | .55 |

*Notes*. M, mean; SD, standard deviation; p_m_, item difficulty; r_it_, corrected item-total correlation. ^a^Item belongs to Weight Concern and Shape Concern.
