# Supplementary material for: How Frequent Are Eating Disturbances in the Population? Norms of the Eating Disorder Examination-Questionnaire
Source: PLoS One. 2012 Jan 18;7(1):e29125. doi: 10.1371/journal.pone.0029125 (PMC3261137; doi:10.1371/journal.pone.0029125)
Supplement: Table S4 — Principal Components Analysis of the Eating Disorder Examination-Questionnaire Items (N = 2520). (DOC) [file pone.0029125.s004.doc]

Table S4. Principal Components Analysis of the Eating Disorder Examination-Questionnaire Items (N = 2520).

|  | EDE-Q | Factors | | |  |
| --- | --- | --- | --- | --- | --- |
|  | Subscale | I | II | III | h^2^ |
| *Unrotated factor solution* |  |  |  |  |  |
| Eigenvalues |  | 10.01 | 2.04 | 1.71 |  |
| % of variance |  | 45.5 | 9.3 | 7.8 |  |
| *Rotated factor solution* |  |  |  |  |  |
| Eigenvalues |  | 5.51 | 4.39 | 3.86 |  |
| % of variance |  | 25.0 | 20.0 | 17.6 |  |
| *Factor loadings after rotation* |  |  |  |  |  |
| Discomfort seeing body | SC | **.84** |  |  | .81 |
| Dissatisfaction with shape | SC | **.84** |  |  | .79 |
| Avoidance of exposure | SC | **.81** |  |  | .74 |
| Dissatisfaction with weight | WC | **.81** |  |  | .75 |
| Importance of shape | SC | **.68** |  |  | .58 |
| Importance of weight | WC | **.68** |  |  | .59 |
| Reaction to prescribed weighing | WC | **.58** |  | .33 | .45 |
| Feelings of fatness | SC | **.58** | .58 |  | .71 |
| Guilt about eating | EC | **.49** |  | .44 | .52 |
| Restraint over eating | RE |  | **.81** |  | .71 |
| Dietary rules | RE |  | **.78** |  | .66 |
| Food avoidance | RE |  | **.77** |  | .68 |
| Desire to lose weight | WC | .54 | **.64** |  | .74 |
| Fear of weight gain | SC | .40 | **.57** | .32 | .59 |
| Flat stomach | SC |  | **.57** |  | .44 |

Table S3 (cont.)

|  | EDE-Q | Factors | | |  |
| --- | --- | --- | --- | --- | --- |
|  | Subscale | I | II | III | h^2^ |
| Preoccupation with food, eating or calories | EC |  | .31 | **.76** | .70 |
| Preoccupation with shape or weight | SC, WC |  |  | **.76** | .67 |
| Social eating | EC | .33 |  | **.71** | .61 |
| Fear of losing control over eating | EC |  | .31 | **.68** | .59 |
| Empty stomach | RE |  | .43 | **.64** | .61 |
| Eating in secret | EC | .33 |  | **.50** | .38 |
| Avoidance of eating | RE |  | .46 | **.50** | .46 |

*Notes.* Principal components analysis (extraction eigenvalues > 1 and scree test) with orthogonal VARIMAX rotation. Only loadings ≥ .30 are displayed. Highest load per item is bolded. EDE-Q, Eating Disorder Examination-Questionnaire; RS, Restraint; EC, Eating Concern; WC, Weight Concern; SC, Shape Concern; h², communalities.
